# A test of the effort equalization hypothesis in children with cerebral palsy who have an asymmetric gait

**DOI:** 10.1371/journal.pone.0262042

**Published:** 2022-01-21

**Authors:** Juha-Pekka Kulmala, Piia Haakana, Jussi Nurminen, Elina Ylitalo, Tuula Niemelä, Essi Marttinen Rossi, Helena Mäenpää, Harri Piitulainen

**Affiliations:** 1 Motion Laboratory, Children’s Hospital, Helsinki University Hospital and University of Helsinki, Helsinki, Finland; 2 JAMK University of Applied Sciences, Jyväskylä, Finland; 3 Faculty of Sport and Health Sciences, University of Jyväskylä, Jyväskylä, Finland; Boston University, UNITED STATES

## Abstract

Healthy people can walk nearly effortlessly thanks to their instinctively adaptive gait patterns that tend to minimize metabolic energy consumption. However, the economy of gait is severely impaired in many neurological disorders such as stroke or cerebral palsy (CP). Moreover, self-selected asymmetry of impaired gait does not seem to unequivocally coincide with the minimal energy cost, suggesting the presence of other adaptive origins. Here, we used hemiparetic CP gait as a model to test the hypothesis that pathological asymmetric gait patterns are chosen to equalize the relative muscle efforts between the affected and unaffected limbs. We determined the relative muscle efforts for the ankle and knee extensors by relating extensor joint moments during gait to maximum moments obtained from all-out hopping reference test. During asymmetric CP gait, the unaffected limb generated greater ankle (1.36±0.15 vs 1.17±0.16 Nm/kg, p = 0.002) and knee (0.74±0.33 vs 0.44±0.19 Nm/kg, p = 0.007) extensor moments compared with the affected limb. Similarly, the maximum moment generation capacity was greater in the unaffected limb versus the affected limb (ankle extensors: 1.81±0.39 Nm/kg vs 1.51±0.34 Nm/kg, p = 0.033; knee extensors: 1.83±0.37 Nm/kg vs 1.34±0.38 Nm/kg, p = 0.021) in our force reference test. As a consequence, no differences were found in the relative efforts between unaffected and affected limb ankle extensors (77±12% vs 80±16%, p = 0.69) and knee extensors (41±17% vs 38±23%, p = 0.54). In conclusion, asymmetric CP gait resulted in similar relative muscle efforts between affected and unaffected limbs. The tendency for effort equalization may thus be an important driver of self-selected gait asymmetry patterns, and consequently advantageous for preventing fatigue of the weaker affected side musculature.

## Introduction

Understanding the key determinants of compensatory gait strategies are of critical importance to our attempts to develop better gait rehabilitation strategies. According to a long-standing *minimum energy cost* hypothesis, the central nervous system (CNS) innately coordinates limb movements so that the resulting gait patterns coincide with the minimal energy expenditure [1,2]. Evidence for this comes primarily from studies on unimpaired locomotion, which have shown that humans (and animals) self-select various gait parameters to minimize their energy expenditure, and even small perturbations to the preferred gait pattern, such as asymmetry, altered step length or width, lead to increases in the energy cost of walking [1–3].

However, the minimum energy cost hypothesis may not apply directly to clinical populations. This is evident from recent studies indicating that self-selected gait asymmetry post-stroke is not unequivocally arising from energy cost minimization [4–6]. Rather, these studies observed that many individuals with impaired gait from chronic stroke were capable of walking more symmetrically without any effect on the energy cost, yet they prefer to walk asymmetrically [4–6]. Stroke often results in muscle weakness and spasticity in the paretic lower limb, and therefore it has been suggested that alternative factors such as perceived effort, avoidance of muscle fatigue, comfort, or stability, could be the principal determinant of certain gait asymmetry patterns [4–6].

The very first requisite for walking is an ability of the antigravity muscles to develop enough force for supporting and propelling the body. Because impaired paretic limb function poses a challenge to meet this requirement, asymmetric gait is considered as an adaptation strategy to the diminished force generation of the affected limb [4–6]. The underlying neural mechanisms behind asymmetric gait adaptation remain unclear but may involve automatic processes which are responsible for sensing and regulating levels of muscle effort associated with the locomotor task. The ‘sense of effort’ is generated centrally in the brain, being based on the proprioceptive afference from the muscles, tendons and joints, and descending motor output [7].

The purpose of this study is to clarify our understanding of the underlying factors behind gait asymmetry. Previous research [4–7] led us to formulate and test an *effort equalization hypothesis*: self-selected gait asymmetry arises from a tendency to equalize the relative muscular efforts between affected and unaffected lower limbs, *i*.*e*., both lower limbs tend to operate at the same proportion of their specific maximal capacity. We used children with hemiplegic cerebral palsy (CP) as a model to study this topic who, similar to individuals with chronic stroke, exhibit asymmetric and metabolically costly walking due to compromised neuromuscular function of the affected side lower limb [8].

## Methods

### Participants

This study was performed as a part of larger project (see details elsewhere [9]), in which 18 spastic hemiplegic and 12 spastic diplegic children with CP underwent 3D gait and hopping analysis in a motion analysis laboratory. Ten of the spastic hemiplegic patients (13.1 ± 2.1 years, 46.9 ± 10.6 kg, 155 ± 12 cm, 9 female) were able to successfully perform the two-leg hopping task, and therefore, were selected for the further analysis. The criterion for acceptable performance was an ability to perform maximal two-leg hopping repeatedly at least three times. All ten CP participants demonstrated the Gross Motor Function Classification System level I, indicating the ability to walk independently but some limited ability to perform gross motor skills [10]. They had no known cognitive or co-operative deficiencies, hearing deficit, visual deficit other than refractive error, orthopaedic surgery done in the lower extremities, condition (other than CP) or medication known to affect gait and balance. The patients were recruited from the rehabilitation unit of the Children’s Hospital, Helsinki University Hospital and by advertising via a patient organization. The study was approved by the ethics committee of Helsinki University hospital (HUS/2318/2016) and was conducted in accordance with the Helsinki Declaration. All the volunteered patients gave informed verbal assent and their guardians gave written informed consent to participate in the study.

### Experiments and analysis

From the three walking conditions (unconstrained gait, motor-dual task-constrained gait, and cognitive dual-task-constrained gait) measured in the larger project, only the normal unconstrained gait with self-selected speed was included in the analysis. After the gait measurement, a two-legged hopping task was performed to quantify the maximum moment for knee and ankle extensors that each participant was able develop during this natural task. Participants were asked to repeatedly hop over two force plates as high as possible at least five times and repeat the hopping task after a 30 s resting period. We selected this plyometric movement as a reference test, because it has been shown to enable humans to produce the greatest extensor muscle moments from the ankle and knee joints [11,12].

For 3D motion analysis, anthropometric measurements (height, weight, leg length, and knee and ankle diameters) were measured for each participant, and 35 retro-reflective markers were placed bilaterally on the participant based on the Plug-in Gait full-body model (Vicon, Oxford, UK). An eighteen-camera system (Vicon T40, Oxford, UK) and four force platforms (AMTI, Watertown, MA) were used to record marker positions and ground reaction force (GRF) data synchronously at 100 and 1000 Hz, respectively. During gait and hopping, three clean force plate contacts of both lower limbs of each participant were selected for the final analysis. To avoid impact artefacts, marker trajectories and GRF data were low-pass filtered using a fourth order Butterworth filter with a cut-off frequency of 18 Hz [13]. Foot contact and toe-off events were detected based on the 10 N vertical GRF threshold level and Plug-in Gait model (Nexus v. 2.09, Vicon, Oxford, UK) was then used to calculate sagittal plane angles and moments for the ankle, knee and hip joints in walking and hopping. Step length was calculated as the anterior-posterior distance between trailing and leading limb toe marker positions defined at the time of initial foot contacts. Data across three full gait and hopping cycles were time normalized (0–100%) and averaged for each participant.

In order to estimate the relative muscle effort levels for the unaffected and affected limbs during gait, we related peak moments generated during gait to maximum moments obtained from the hopping task [12,14]. This relative effort value indicated how close ankle and knee extensors operated to their maximal moment generation capacities during walking.

### Statistical analysis

Statistical tests were performed with IBM SPSS software (Version 25.0, Chicago, IL, USA). Shapiro-Wilk test was used to confirm the normal distribution and paired two-tailed t-test was then used to examine whether relative efforts differed between affected and unaffected limb ankle and knee extensors. In addition, to further understand side-to-side asymmetries during gait and hopping task, statistical analysis with paired two-tailed t-test was also performed for selected spatio-temporal (step length and stance time), kinematic (joint angles and angular velocities), and kinetic (peak vertical GRF and joint moments) variables. For the three variables that were not normally distributed (ankle joint angle at peak moment during gait, knee joint angle at peak moment during hopping, and ankle joint angular velocity at peak moment), the Wilcoxon Signed Rank test was used for statistical comparison. p < 0.05 was considered significant.

## Results

### Joint moments during gait

**Fig 1:**
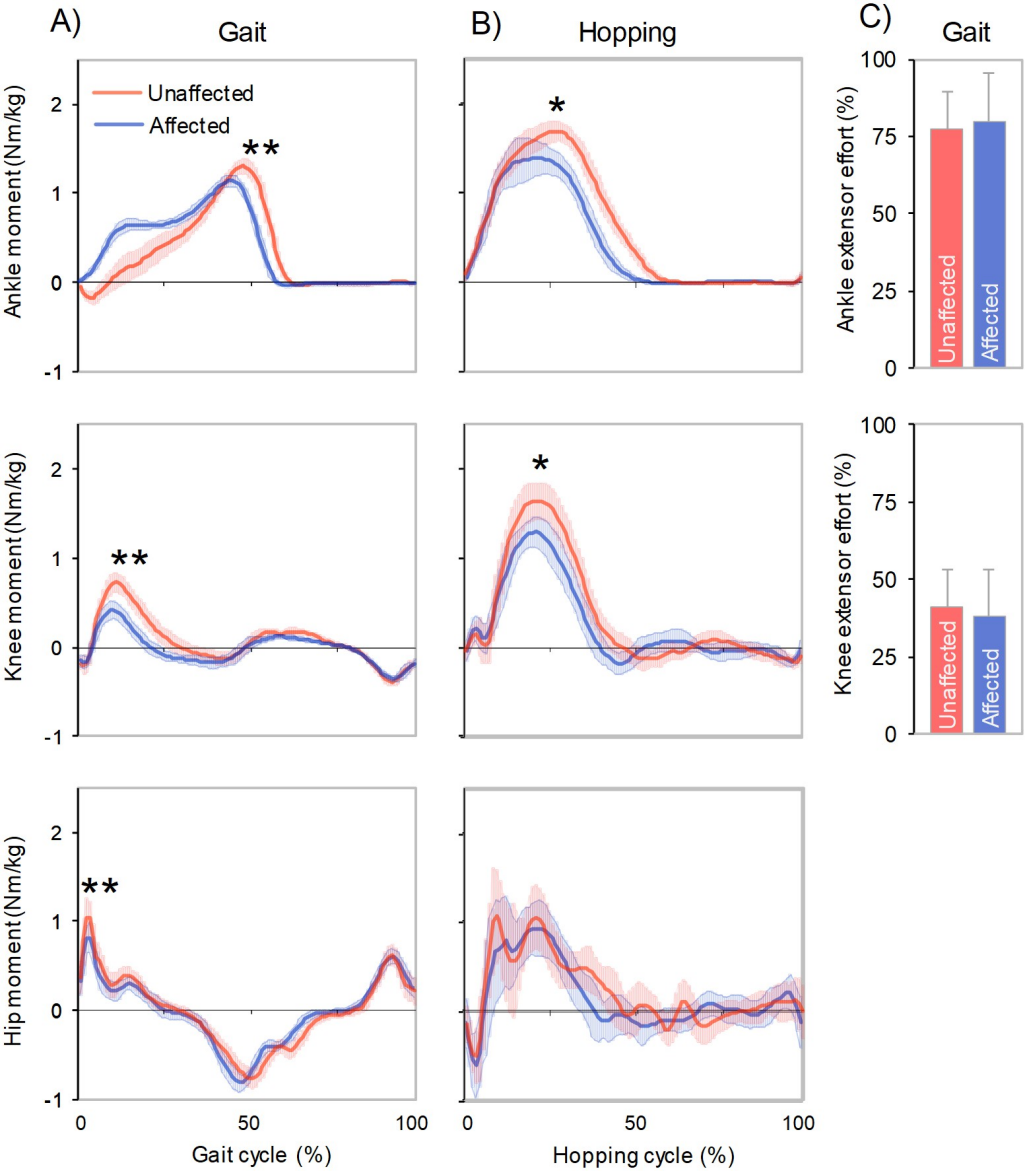
Lower limb joint moments across limbs during A) gait and B) two-leg hopping test. C) Relative efforts for the ankle and knee extensors during gait.

Children with hemiplegic CP walked at speed 1.22 ± 0.08 m/s. We observed 0.04 s shorter stance time (p = 0.007) and a tendency towards smaller step length (2.1 cm, p = 0.1) in the affected limb compared to unaffected limb (Table 1). A notable gait asymmetry was present in the limb extensor moments, where 16% (p = 0.002), 68% (p = 0.007) and 25% (p = 0.020) greater values were found at the unaffected ankle, knee and hip, respectively, compared to the affected limb (Fig 1, Table 1).

**Table 1:** Comparisons of the spatio-temporal, kinematic and kinetic data across limbs during the gait and hopping test.

	Gait			Hopping		
	Unaffected	Affected	*t*-test	Unaffected	Affected	*t*-test
Spatio-temporal & GRF						
Step length (cm)	60.6 ± 3.5	58.5 ± 3.6	0.100			
Stance time (s)	0.59 ± 0.06	0.55 ± 0.06	**0.007**	0.31 ± 0.05	0.28 ± 0.03	**0.015**
1^st^ peak vertical GRF (BW)	1.16 ± 0.14	1.15 ± 0.08	0.728	1.79 ± 0.26	1.43 ± 0.24	**0.019**
Peak joint moments (Nm/kg)						
Ankle extensors	1.36 ± 0.15	1.17 ± 0.16	**0.002**	1.81 ± 0.39	1.51 ± 0.34	**0.033**
Knee extensors	0.74 ± 0.33	0.44 ± 0.19	**0.007**	1.83 ± 0.37	1.34 ± 0.38	**0.021**
Hip extensors	1.19 ± 0.27	0.95 ± 0.22	**0.020**	1.61 ± 0.35	1.44 ± 0.53	0.501
Relative effort (%)						
Ankle extensors	77.4 ± 12.4	80.1 ± 15.5	0.692			
knee extensors	41.0 ± 17.3	38.0 ± 23.2	0.541			
Joint angle at peak moment (deg)						
Ankle	13.5 ±4.3	11.7 ± 5.2	0.386	30.6 ± 4.5	21.0 ± 11.0	**0.041**
Knee	24.3 ± 6.2	21.2 ± 5.8	0.113	61.1 ± 7.1	65.1 ± 6.7	**0.028**
Joint angular velocity at peak moment (degs^-1^)						
Ankle	46.3 ± 21.6	19.1 ± 20.2	**0.003**	109.6 ± 72.0	33.3 ± 40.6	**0.037**
Knee	-1.8 ± 26.4	-0.4 ± 29.1	0.930	37.9 ± 65.9	43.1 ± 47.8	0.847

Data shown as mean ± s.d. BW, body weight.

Positive angular velocities indicate shortening of the muscle-tendon units at peak moment while negative values represent lengthening of the muscle-tendon units at peak moment.

### Joint moments during hopping

During all-out hopping test, the maximal moments developed by the unaffected side ankle and knee extensors were 17% (p = 0.033) and 36% (p = 0.021) greater, respectively, than in the affected side (Fig 1, Table 1). Also, the total limb force production, determined as a peak vertical GRF during the contact phase, was 25% (p = 0.019) greater in unaffected versus affected limb.

### Relative ankle and knee extensor muscle efforts during gait

**Fig 2:**
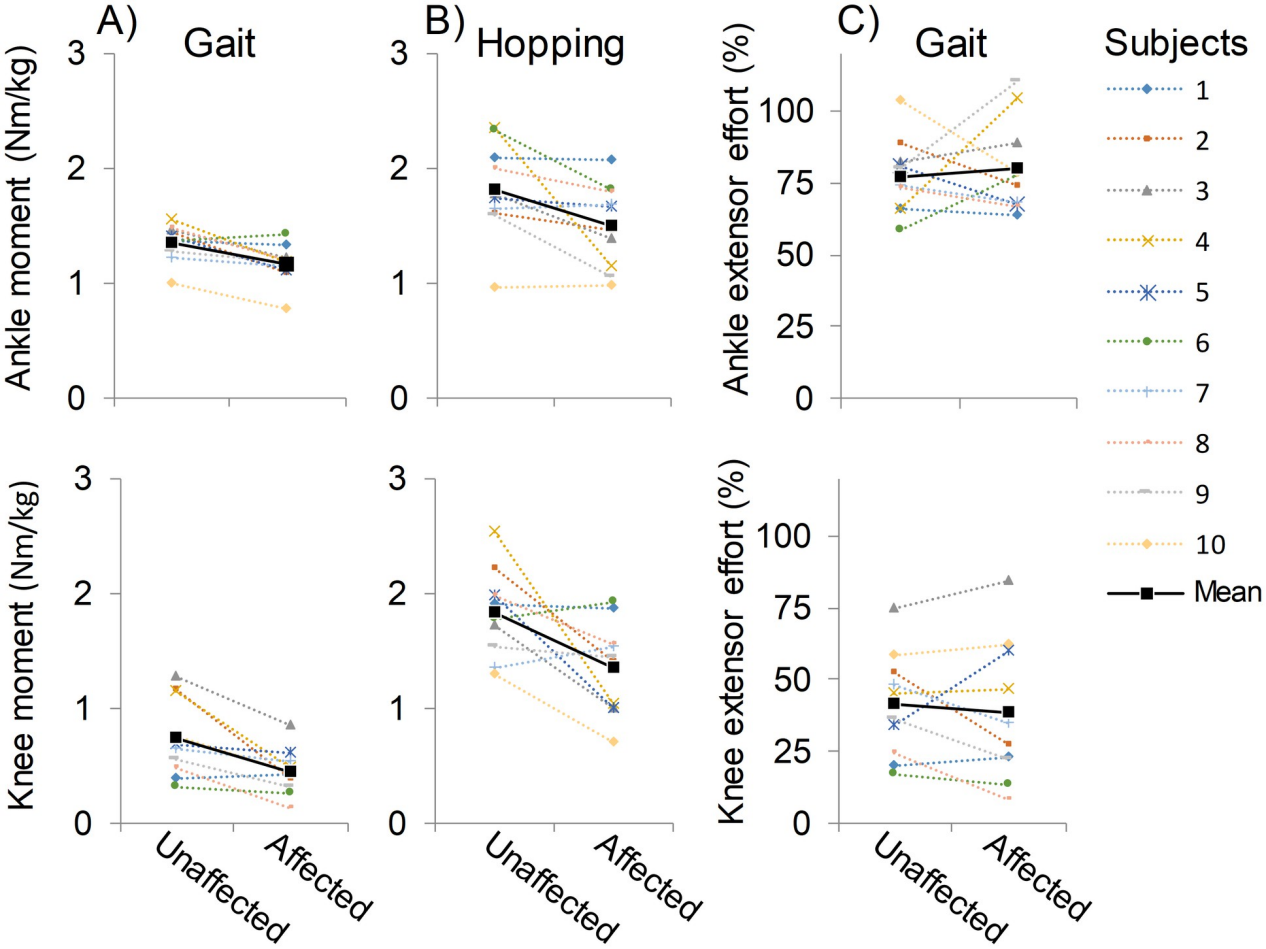
Individual peak joint moments during A) gait and B) hopping test, and C) relative muscle efforts during gait.

The analysis of relative muscle effort showed no differences for the ankle (p = 0.692) and knee (p = 0.541) extensors between affected and unaffected limbs during gait (Fig 1, Table 1). On average, the ankle extensors on both sides operated with two times greater efforts compared to the knee extensors during gait, although clear individual differences were observed within both muscle groups (Fig 2).

## Discussion

In line with our effort equalization hypothesis, children with hemiplegic CP demonstrated similar relative ankle and knee extensor muscle effort levels in the affected and unaffected lower limbs during gait, even though these muscle groups were much weaker in the affected limb in our hopping reference test. To achieve similar relative efforts between limbs during walking, the participants generated greater ankle and knee extensor moments in the unaffected versus the affected limb. These kinetic changes were accompanied by clear stance time asymmetry, being well in line with previous literature on hemiparetic gait due to CP.

Consequently, the present findings support the idea that self-selected gait asymmetry patterns in clinical populations may arise primarily from a tendency to equalize relative muscle efforts between affected and unaffected limbs. This, together with previous work [4–6], suggests that the preferred gait patterns in clinical populations are not purely determined by energy cost minimization but are greatly influenced by others factors like constraints imposed by task demands and available muscular capacity. As marked unilateral weakness characterize individuals with hemiparetic gait [8,15], the affected limb muscles may be particularly susceptible to an early-onset fatigue during walking. Thus, although the self-selected asymmetric patterns of hemiparetic gait, such as reduced extensor muscle moment generation in the affected versus unaffected limb, may not provide metabolic benefits beyond a more symmetric gait pattern, these patterns may be advantageous in optimizing prolonged walking performance by preventing fatigue of the weaker affected limb muscles.

The ‘sense of muscle effort’ can be an important neuronal mechanism in the effort equalization phenomenon. The sense of muscle effort that is generated in the brain based on proprioceptive afferent information from the locomotor system, is specific to each limb and joint underlying its adaptability, and is known to affect the efferent motor command from the brain to the muscles [7]. This sense of effort is closely associated, if not equal, with the functional range of moment output in a given joint, and thus the joint moment outputs could be optimized at relative scale with respect to the maximal effort predicted by the brain [7]. The effort should not be too high to avoid excessive fatigue but should meet the given task demands and functional constraints of the locomotor system to support the aim and efficiency of the motor task. In the case of CP, the peripheral motor impairment may adjust the ‘sense of effort’ and the related motor output from the brain separately for the paretic and non-paretic limbs. This modulation is expected to happen mainly in the brain, since the function of the proprioceptive afference pathways from lower limbs to the brain do not seem to be dramatically affected in adolescents with CP who are able to walk without assistive devices [16]. Piitulainen et al. [16] showed using magnetoencephalography (MEG) and proprioceptive stimulation of the ankle joint, that the primary somatosensory cortex response magnitude (i.e. cortical activation) does not differ between paretic and non-paretic limbs, or when compared to typically developed healthy peers.

Notably, locomotor adaptations to equalize muscle efforts have also been noted among the healthy population. The phenomenon called distal-to-proximal shift in joint kinetics is a well-recognised adaptation mechanism in older adults’ gait [17,18] and is also noted to occur in runner’s gait due to fatigue [19,20]. Specifically, in this gait adaptation, the kinetic contribution from the ankle joint during stance phase is progressively reduced and replaced by a greater contribution from more proximal knee and/or hip joint musculature [17–20]. Previous studies have attributed this distal-to-proximal shift adaptation mechanism to the fact that both walking and running require greater relative efforts from the ankle extensors compared to more proximal lower limb muscle groups [12,14,21,22], thereby indicating a lower force reserve at the ankle extensors to buffer any loss of muscle capacity that may occur due to ageing, fatigue or disease. It thus seems likely that redistribution in muscular contributions, either between or within limbs, are two aspects of the same effort equalization principle that may help to optimize locomotor performance.

In line with previous studies on relative muscle efforts [12,14,21,22], the ankle extensors in children with hemiplegic CP showed about two times greater relative efforts compared to the knee extensors during gait. Although no direct evidence for the distal-to-proximal shift phenomenon in clinical populations has yet appeared, the great demand of the ankle extensors suggests their vulnerability to fatigue during prolonged walking and consequent appearance of the distal-to-proximal shift compensation strategy. Moreover, a previous study [23] using electromyographic (EMG) analysis found fatigue related reduction in median frequency of EMG and increase in the EMG amplitude at the gastrocnemius medialis and soleus that was not observed in the more proximal muscles, indicating a greater level of fatigue in the distal muscles (i.e. the ankle extensors). Given this notion together with the present evidence, there is reason to believe that ankle extensor fatigue may become a key limiting factor of prolonged walking performance in children with hemiplegic CP.

The current findings provide some new insight on the persistence of gait abnormalities in children with CP even after intensive treatment modalities. For example, a large recent study of Shuman et al. (2019) indicated that neuromuscular gait coordination is essentially unchanged in children with CP, and their gait speed is reduced rather than increased despite comprehensive treatments (either botulinum toxin type A injections, selective dorsal rhizotomy, or multi-level orthopedic surgery) and intensive rehabilitation [24]. The authors therefore inferred that children with CP exhibit persistent fixed motor control strategy of gait, which they attributed to limited neural plasticity caused by injury in the immature brain [24]. However, at least to some extent, limited outcomes in gait speed and coordination after these treatments can also be due to patients’ limited ability to strengthen the weak and spastic CP muscles [25,26]. Indeed, accumulating evidence suggests that reduced muscle strength is the principal factor constraining gait in CP [27,28]. Furthermore, strength improvements following interventions appear to relate to the achievement of positive outcomes [29,30]. For example, an intervention-induced increase in ankle extensor strength in a recent gait training study was strongly related to the improvements in ankle push-off intensity and better symmetry during gait in children with CP [31]. Taking these findings together, it can perhaps be argued that if the individuals find it preferable to avoid high muscle efforts during gait, and if the muscular capacity remains unchanged following treatment, it is plausible that the individuals continue to rely on the same compensatory gait strategies in order to ensure joint stability against the force of gravity.

This study has important limitations that need to be considered. First, the participants in this study were children with hemiplegic CP but previous observations that the self-selected asymmetry of hemiparetic gait does not unequivocally coincide with the minimal energy cost have been made in individual with chronic stroke [4–6]. Therefore, caution should be exercised in generalizing these results to patient groups other than hemiplegic CP. Second, although we included only participants who successfully performed our hopping test, we cannot completely confirm that the maximum moment generation capacity was reached by all participants. In particular, one could expect this to be true in the affected limb, which would lead to a systematically smaller maximum moment values and consequently increased muscle efforts in this limb. However, this does not appear to be the case since the individual data in Fig 2 show non-systematic variation in the hopping-related maximum joint moments between affected versus unaffected limb. Specifically, this variation may indicate that some participants did not reach their maximum moment generation in the affected limb and others in the unaffected limb. Importantly, however, in the affected limb our participants exhibited maximal ankle and knee extensor moments that were 83% and 74%, respectively, of the values in the unaffected limb, which fairly well match to previous dynamometer-derived results in the literature, where maximal affected limb ankle and knee extensor strength values are 50–77% and 62–87%, respectively, of those of the unaffected limb values [27,32,33]. This provides us with confidence that the hopping test generally allowed a reliable measurement of the maximal moment output and consequent determination of relative muscle effort for both limbs. Third, the inverse dynamics–based joint moment analysis was unable to account for the effects of muscle co-contractions, two-joint muscles and passive properties of the joint [34]. Again, these issues may limit the accuracy of determining the muscle efforts especially at the affected limb ankle extensors in children with CP because of their potentially greater antagonist muscle co-contraction and restrictions of passive ankle dorsiflexion movement [35]. However, because gait and hopping are both affected, it is unlikely that our primary findings are significantly influenced by this issue. Fourth, our hopping reference test did not allow us to quantify maximal capacity and thus operating efforts of the hip extensors and abductors, which also have an important role in the achievement of normal gait. And finally, we were unable to perfectly match the muscle contractile conditions (joint angles and angular velocities) between the gait and hopping tasks. We note that during gait the lower limb is more extended and the advantage from stretch-shortening cycle on muscle force generation is less pronounced [36]. Although these differences limit the accuracy with which we can estimate the operating muscle efforts in this study, the relatively low angular velocities of both the knee and ankle joints at the time of peak joint moment were observed in gait and hopping (Table 1), suggesting that muscle contractile conditions remain nearly isometric, and thus, favorable for producing force in both activities [37–40]. An isometric inverse dynamics-based test pattern for determining the maximal joint moments at locomotion-matched joint angles has the potential to overcome some of these limitations in the future [41]. Such a test pattern would be easier to perform and, therefore, could be more suitable when attempting to quantify muscular efforts in individuals with limited functional abilities.

## Conclusion

This study provides support for the effort equalization hypothesis that through asymmetric gait, children with hemiplegic CP equalize relative muscle efforts between their affected and unaffected limb ankle and knee extensors. Therefore, it seems likely that in addition to energy cost minimization [1–3], a tendency to equalize muscle efforts, either between or within limbs, is a key determining factor behind preferred gait patterns. These insights have relevance for the understanding and treatment of gait asymmetries in clinical populations.

## Supporting information

S1 Data (XLSX)
